# Sharing the Details: Implementing and Evaluating the Integration of New York State AIDS Institute Health Equity Competencies for Health Care Providers into Clinical Training Activities

**DOI:** 10.1089/heq.2023.0123

**Published:** 2023-09-13

**Authors:** Brooke A. Levandowski, Hannah R. Murphy, Jessica Silk, Cabiria M. Barbosu, Marguerite Urban, Lauren Walker, Beatrice Aladin, Timothy D. Dye

**Affiliations:** ^1^ Department of Obstetrics and Gynecology, University of Rochester, Rochester, New York, USA.; ^2^ Institute for Advanced Medicine, Mount Sinai Health System, New York City, New York, USA.; ^3^ Department of Medicine, University of Rochester, Rochester, New York, USA.; ^4^ New York State Department of Health AIDS Institute, Albany, New York, USA.

**Keywords:** health equity, program evaluation, education, continuing, HIV, sexually transmitted diseases, prevention & control

## Abstract

**Background:**

The New York State (NYS) Department of Health (DOH) AIDS Institute (AI) Clinical Education Initiative (CEI) trains the NYS health care workforce to improve health outcomes related to HIV, sexual health, hepatitis C, and for people who use drugs.

**Methods:**

In 2019, CEI began consistently integrating health equity into CEI activities through a working group that mapped NYS DOH AI health equity competencies for providers onto planned clinical education. We conducted a convergent mixed methods study on qualitative and quantitative participant feedback form (PFF) data to evaluate these competencies between April 1, 2021, and September 30, 2022, and conducted an annual survey of NYS clinician needs in 2021 and 2022.

**Results:**

The CEI Health Equity Working Group analyzed 25 measures within 4 health equity competencies that were grouped into 4 interventions: resources, internal tools, activity creation, and evaluation. Eighty-nine percent of PFF respondents (*n*=20,166) strongly agreed/agreed that CEI activities included multiple viewpoints; qualitative comments described informative and helpful activities. When asked how they address patient-identified social determinants of health (SDOH) needs, 84% and 71% of annual survey respondents reported they made the highest number of referrals for health insurance coverage assistance in 2021 and 2022, respectively.

**Discussion:**

CEI continues to address participant feedback and seamless incorporation of health equity components into their work.

**Health Equity Implications:**

Health equity in clinical practice and trainings is crucial in acknowledging and addressing SDOH that continue to impact NYS clinicians and their patients.

## Background

Many institutions have supported addressing systematic injustices, disparities, and inequalities, particularly in health care outcomes and related social determinants, through a health equity approach.^1–3^ Health equity refers to fair and equal opportunities for all to maintain health while removing systemic obstacles of discrimination, poverty, and related downstream effects.^4^ Although not a new concept, addressing health inequities has gained significant traction in recent years with a focus on clinicians (including physicians, nurses, and other health care professions) providing equitable, nondiscriminatory, and affirming services.^2,5,6^

Organizations such as the Centers for Disease Control and Prevention's Healthy Communities Program, The Health Equity Initiative, the Association of American Medical Colleges, and national nursing organizations (including the American Association of Colleges of Nursing, American College of Nurse-Midwives, and American Nurses Association) support a variety of equity, diversity, and inclusion initiatives.^7–9^

The New York State (NYS) Department of Health (DOH) AIDS Institute (AI)'s Clinical Education Initiative (CEI) is designed to enhance the capacity of NYS's diverse health care workforce to deliver clinical services and to improve health outcomes related to HIV, sexually transmitted infections (STIs), hepatitis C (HCV), for people who use drugs, and emerging health issues.

CEI has four aims: (1) provide progressive HIV, HCV, STIs, and clinical care for people who use drugs-related education to clinicians, including free continuing medical education, continuing nursing education, and continuing pharmacy education credits; (2) disseminate NYSDOH AI clinical practice guidelines; (3) expand the base of clinicians able to diagnose and care for HIV, HCV, and STI patients and people who use drugs; and (4) foster partnerships between community-based clinicians and HIV, HCV, STI specialists, and experts working with people who use drugs. CEI provides live (e.g., webinars, conferences, preceptorships, and training series) and online trainings to NYS clinicians (including physicians, advanced practice providers, pharmacists, nurses, dentists, and nurse midwives) and offers >400 accredited courses.

Through comprehensive training and services, CEI addresses key social determinants of health (SDOH) that impact the delivery of health care and the populations served. Reaching and providing services to historically excluded groups remain a driving force behind the implementation of the CEI aims. However, reaching historically excluded populations is only one aspect of appropriately trained clinicians. Clinical training must also reflect upon how SDOH impact patients.^10^ Equitable health services rely upon the acknowledgment and active practice of antiracist, culturally appropriate, and gender-affirming services, and it is important that these topics are adequately presented in clinical training.

Since 2019, CEI has intentionally added a health equity lens to the clinical education offered on HIV/HCV/STI screening/treatment/prevention and clinical care for people who use drugs. The focus on health equity is expressly promoted by the AI^11^ and the Health Competencies for Health Care Providers^12^ guidelines created in 2021.

Evaluating CEI participants' experiences since the intentional implementation of this health equity lens is crucial to continue to improve equity in the provision of services. Following each CEI activity, participants are asked to evaluate program metrics such as format, speaker knowledge, usefulness, bias mitigation, and knowledge gained. Participant feedback is a crucial mechanism for improving and expanding CEI trainings to better serve NYS clinicians.

## Methods

### Implementation

In 2021, the NYS DOH AI convened the Health Equity Competencies Clinical Work Group, which developed Health Competencies for Health Care Providers.^7^ Their competencies included four areas: (1) addresses SDOH, (2) takes an active role in community and institution, (3) employs a person-centered model of care, and (4) seeks to avoid bias and provides affirming services. The document includes several competencies under each category. Clinicians are encouraged to review each competency, think about the realities faced by their patient population, and determine which competencies they can immediately address, and which competencies would require further support.

For example, examples of competencies in the person-centered model of care include (1) uses clear communication to convey health information and (2) makes time to listen and actively supports patient self-determination using an autonomy-supportive approach. Every patient encounter is not expected to address all of the competencies, yet clinicians are encouraged to actively integrate competencies into care provision.

The CEI Health Equity Working Group analyzed health equity competencies using the following process: (1) reviewed all four categories and the competencies under each category, (2) determined whether CEI could address the competency at the clinician training level or whether it required organizational support, and (3) determined how or whether CEI could directly support the competency.

### Evaluation

We conducted a convergent mixed methods study using qualitative and quantitative data captured between April 1, 2021, and September 30, 2022. Every CEI activity is followed by emailing a link to a participant feedback form (PFF) to all participants. PFF completion is required of all participants who requested continuing education credits. Beginning April 1, 2021, we evaluated health equity efforts by adding an optional statement, “This activity included multiple points of view (related to race, ethnicity, gender identity, sexual orientation, etc.) and included information that could improve health equity among the patients served,” with a 5-point Likert scale ranging from “strongly agree” to “disagree.”

We conducted chi-square tests of significance (Stata v16.1). We completed qualitative analysis using inductive coding to identify main themes, which was conducted separately by two authors (B.A.L. and H.R.M.) and agreed upon together. We conducted mixed methods convergent analyses by integrating quantitative results with qualitative responses from the final open-ended question asking for any additional feedback.^13^

To support our efforts to improve trainings offered and resources provided, CEI also conducts an annual survey to better understand clinicians' knowledge, practice, needs relating to HIV, HCV, and STIs, providing clinical care to people who use drugs, and priority topics such as COVID-19 and emerging topics that may impact trainings and educational activities provided to clinicians. CEI sent a REDCap survey link to former and current CEI audiences in 2021 and 2022 through announcements in a special edition of the CEI newsletter, regular CEI newsletters, the website, mobile site, CEI Facebook, CEI Instagram, and CEI LinkedIn. We conducted descriptive statistics (Stata v16.1).

This evaluation was conducted within the CEI Program and Evaluation Wave II protocol that was determined as not human research by the University of Rochester Research Subjects Review Board (STUDY00006249).

## Results

### Implementation

The CEI Health Equity Working Group analyzed 25 measures within four health equity competencies that were grouped into four interventions: (1) create a repository of resources for clinicians and health care organizations to support health equity competencies, (2) create internal tools to integrate health equity competencies across programming, (3) develop activities to support health equity competencies as they relate to CEI content areas (HIV, STIs, HCV, and clinical care for people who use drugs), and (4) integrate health equity into program evaluation activities. The working group then developed year-long work plans, focused on developing one intervention per quarter, beginning October 1, 2021.

Based on the working group findings, CEI updated and expanded the resources section of the CEI website, developed internal tools to assist program planners and faculty with incorporating the Health Competencies for Health Care Providers, produced health equity-focused programming related to CEI content areas, and integrated health equity into program evaluation (Table 1).^12^

**Table 1.** Health Equity Products Developed and Implemented by Clinical Education Initiative in Response to Four Health Equity Interventions

Health equity intervention	CEI products
Create a repository of resources for clinicians and health care organizations to support health equity competencies	The health equity resources page of the CEI website provides links to health equity, SDOH, avoiding bias and providing affirming services, person-centered care, and active role in community and institution resources.
Create internal tools to integrate health equity competencies across programming	**Health Equity Guiding Principles** document offers CEI faculty and staff guidance for how to “bake” health equity into CEI's work. Shared principles help send clear and consistent messages to participants. **Health Equity Guidelines for Faculty** document includes shared health equity terminology and examples of correctly using health equity in curricula. **Health Equity Acknowledgment** slide is used for all CEI presentations to show CEI's commitment and to encourage health equity-specific evaluation feedback.
Develop activities to support health equity competencies as they relate to CEI content areas (HIV, STI, HCV, and clinical care for people who use drugs)	CEI centers create health equity-specific content across topic areas. Examples include: “Caring for pregnant persons with substance use disorder: shifting from criminalization to chronic disease management” “Common roots: racism and parallels between COVID-19 and HIV/STI inequities” “Advancing health equity in HIV prevention and treatment: the NYS HIV primary care and prevention annual conference”
Integrate health equity into program evaluation activities	A health equity question was added to the PFF required for continuing education credits. Health equity competency questions were added to the annual survey.

CEI, Clinical Education Initiative; HCV, hepatitis C; PFF, participant feedback form; SDOH, social determinants of health; NYS, New York State.

CEI developed health equity-specific courses including “Antiracism and Harm Reduction,” “HIV, Substance Use, and Social Justice,” “Antiracism and Harm Reduction,” “PrEP, Sexual Health, and Recently Incarcerated Women,” “Social Justice and HIV,” “Engaging LGBTQ+ People who Use Drugs: Cultural Responsiveness and Beyond,” and “Transgender-Affirming Primary Care.”

CEI activities also included longer (5–8 h) programs with integrated health equity-related themes, attracting participation of many NYS clinicians. Examples include the 2022 NYS HIV Primary Care and Prevention Annual Conference: “Advancing the HIV Care Continuum: From Routine Testing to Optimizing Patient Outcomes,” the 8th Annual NYS Sexual Health Conference: “Directions, Perspectives, and Predictions,” and the 2022 Annual NYS Hepatitis C and Drug User Health Conference: “The Next Stage in Hepatitis C and Drug User Health Care: New Approaches for New York State Clinicians.”

### Evaluation

**FIG. 1.**
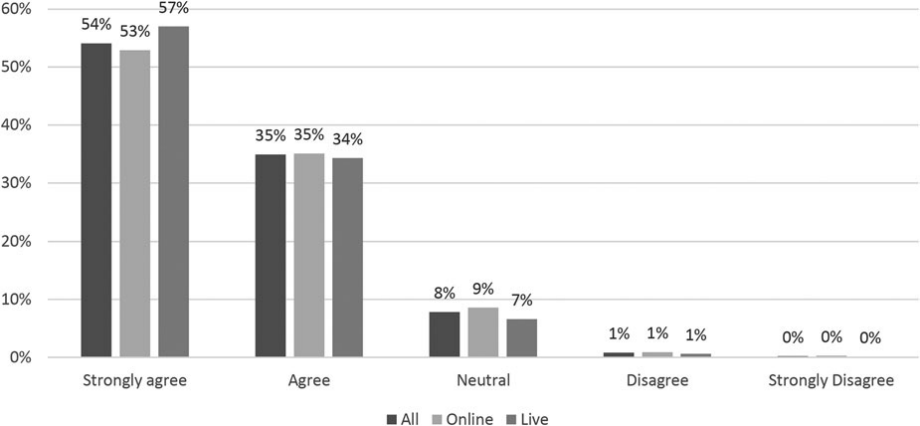
Evaluation of CEI activities using an evaluation question that indicated the “activity included multiple points of view (related to race, ethnicity, gender identity, sexual orientation, etc.) and included information that could improve health equity among the patients I serve including health equity concepts,” April 1, 2021,–September 30, 2022. CEI, Clinical Education Initiative.

In total, 23,085 PFFs were completed between April 1, 2021, and September 30, 2022, of which 30.9% (*n*=7132) participated in live activities through webinar and 69.1% (*n*=15,953) participated in online activities. Each PFF was counted singly, appreciating many clinicians participate in several activities each year. In total, 98.1% (*n*=22,648) responded to the health equity question. Among all respondents, 54.1% strongly agreed and 34.9% agreed that the training activity included health equity concepts and felt the activity included multiple points of view, including information that could improve health equity among their patient population (Fig. 1). There was no statistically significant difference in responses to this question based on participation in online or live activities (*p*=0.735).

Of the 587 qualitative responses given to online activities, 24.9% were substantive. Of the 464 qualitative answers given for live activities, 32.8% included answers beyond “none” or “not applicable.” The vast majority of comments were positive, indicating the activity was informative and helpful. Six total answers specifically mentioned health equity concepts: three from online and three from live activities. Participants suggested exploring other SDOH, digging deeper into socioeconomic status, and adding a “trans man viewpoint.” One participant questioned whether a speaker had used an incorrect term to address a historically excluded population, which has since been addressed with the speaker.

The remaining two comments from each activity type were positive, with one commenting on a live activity by saying, “The presenter made a useful and culturally appropriate presentation from which I learned much. Thank you!” Clinicians who reported positive qualitative health equity comments strongly agreed or agreed with the statement asking about the inclusion of health equity concepts.

**FIG. 2.**
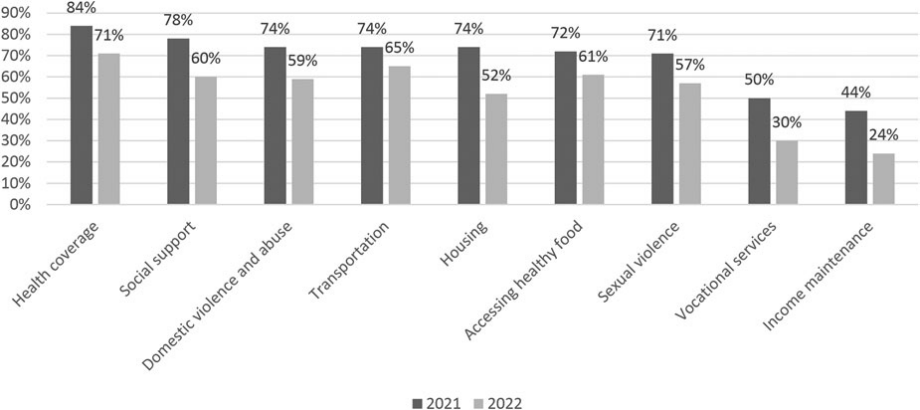
Evaluation of CEI activities using an annual survey question asking which type of referrals clinicians make to community resources for patient assistance, 2021 and 2022.

In July 2021, the CEI NYS Annual Survey was completed by 147 respondents (including 125 clinicians) and in 2022, by 241 respondents (including 192 clinicians). Clinicians noted that referrals addressing individually identified SDOH needs were most often for health insurance coverage (2021, 84%; 2022, 71%), followed by transportation needs (2021, 74%; 2022, 65%), accessing healthy foods (2021, 72%; 2022, 61%), social support (2021, 78%; 2022, 60%), and domestic violence and abuse (2021, 74%; 2022, 59%) (Fig. 2). Clinicians noted active collaborations with care managers (2021, 85%; 2022, 67%), community health workers (2021, 74%; 2022, 63%), and peer workers (2021, 68%; 2022, 43%).

In 2021, the majority of clinicians (69%) reported some form of implicit bias training in the past year, compared with 48% in 2022. Of those who reported having had training, much fewer reported that they actually employed tools for examining implicit bias in their practice (27% in 2021; 20% in 2022). Questions examined various models of care intended to address SDOH for each patient, and which of those models of care had been integrated into practice by annual survey clinicians. Motivational interviewing was most utilized (2021, 71%; 2022, 60%), followed by harm reduction (2021, 65%; 2022, 55%), and trauma-informed care (2021, 52%; 2022, 48%).

## Discussion

The PFF was useful in collecting information on how CEI activities were addressing health equity. The majority (89%) of the respondents reported that CEI activities included information to improve health equity. To address the limitation of potentially low response rates, CEI made this question required to increase data capture moving forward. CEI processes allow each content center of excellence (COE) that produced the activity to easily download a summary of the PFF, with qualitative responses included. The open-ended question is especially useful in capturing opportunities for improvement, such as the one presenter's language, indicated above.

This immediate feedback loop for evaluation data allows content COEs to quickly identify and address participant concerns. CEI will continue to refine activities to capture input on health equity and use evaluation information to address presenters' challenges, creating more equitable presentations that appropriately include historically excluded populations in sensitive ways.

With ∼50% of an individual's health overall being actively impacted by the physical environment and socioeconomic factors, it is concerning that clinical trainings still often lack core competencies in addressing health equity.^14,15^ Studies have shown that exposure to health equity in clinical training leads to enhanced education and clinical experience for both clinicians and those they serve.^16^ Health care systems, education providers, and clinicians all must take responsibility to include equity trainings and policies to best support their historically excluded patient populations.^17^

## Health Equity Implications

Health equity in clinical practice and trainings is crucial in acknowledging and addressing SDOH that continue to impact NYS clinicians and their patients. In 2014, NYS announced a three-point plan to end the AIDS epidemic within the state, with the goal of achieving the first-ever decrease in HIV prevalence by the end of 2020.^18^ NYS adhered to the National Strategic Plan's goals to reduce new HIV infections by 90% by 2030.^19^ The strategic plan includes many references to reducing stigma, discrimination, and adopting a health equity lens, particularly among HIV clinicians.^20,21^

Similar efforts have been implemented to address health disparities related to STIs and people who use drugs, with a focus on harm-reduction strategies among clinicians.^22–24^ Through intentional health equity inclusion in all live and online trainings, CEI is dedicated to enhancing clinical services related to HIV, STIs, HCV, and clinical care for people who use drugs. As clinicians and trainers include more health equity concepts into their work, these evaluation results are important to health practice, as a reminder of the importance of incorporating evaluation feedback into continuous improvement of programming.

## Abbreviations Used

AI: AIDS Institute
CEI: Clinical Education Initiative
COE: center of excellence
DOH: Department of Health
HCV: hepatitis C
NYS: New York State
PFF: participant feedback form
SDOH: social determinants of health
STIs: sexually transmitted infections
